# Attenuated Food Anticipatory Activity and Abnormal Circadian Locomotor Rhythms in *Rgs16* Knockdown Mice

**DOI:** 10.1371/journal.pone.0017655

**Published:** 2011-03-09

**Authors:** Naoto Hayasaka, Kazuyuki Aoki, Saori Kinoshita, Shoutaroh Yamaguchi, John K. Wakefield, Sachiyo Tsuji-Kawahara, Kazumasa Horikawa, Hiroshi Ikegami, Shigeharu Wakana, Takamichi Murakami, Ram Ramabhadran, Masaaki Miyazawa, Shigenobu Shibata

**Affiliations:** 1 Department of Anatomy and Neurobiology, Kinki University School of Medicine, Osaka-Sayama, Osaka, Japan; 2 Department of Physiology and Pharmacology, School of Advanced Science and Engineering, Waseda University, Shinjuku-ku, Tokyo, Japan; 3 Department of Immunology, Kinki University School of Medicine, Osaka-Sayama, Osaka, Japan; 4 Tranzyme Pharma, Durham, North Carolina, United States of America; 5 Department of Endocrinology, Metabolism and Disease, Kinki University School of Medicine, Osaka-Sayama, Osaka, Japan; 6 Technology and Development Team for Mouse Phenotype Analysis: Japan Mouse Clinic, RIKEN Bioresource Center, Tsukuba, Ibaraki, Japan; 7 Department of Radiology, Kinki University School of Medicine, Osaka-Sayama, Osaka, Japan; Pennsylvania State University, United States of America

## Abstract

Regulators of G protein signaling (RGS) are a multi-functional protein family, which functions in part as GTPase-activating proteins (GAPs) of G protein α-subunits to terminate G protein signaling. Previous studies have demonstrated that the *Rgs16* transcripts exhibit robust circadian rhythms both in the suprachiasmatic nucleus (SCN), the master circadian light-entrainable oscillator (LEO) of the hypothalamus, and in the liver. To investigate the role of RGS16 in the circadian clock *in vivo*, we generated two independent transgenic mouse lines using lentiviral vectors expressing short hairpin RNA (shRNA) targeting the *Rgs16* mRNA. The knockdown mice demonstrated significantly shorter free-running period of locomotor activity rhythms and reduced total activity as compared to the wild-type siblings. In addition, when feeding was restricted during the daytime, food-entrainable oscillator (FEO)-driven elevated food-anticipatory activity (FAA) observed prior to the scheduled feeding time was significantly attenuated in the knockdown mice. Whereas the restricted feeding phase-advanced the rhythmic expression of the *Per2* clock gene in liver and thalamus in the wild-type animals, the above phase shift was not observed in the knockdown mice. This is the first *in vivo* demonstration that a common regulator of G protein signaling is involved in the two separate, but interactive circadian timing systems, LEO and FEO. The present study also suggests that liver and/or thalamus regulate the food-entrained circadian behavior through G protein-mediated signal transduction pathway(s).

## Introduction

The circadian timing system in mammals exerts control over a wide range of physiology and behavior, including daily environmental changes, the circadian system can be reset by external time cues (Zeitgeber) such as light and metabolic correlates of feeding [1]–[3]. Two separate, but coupled oscillators, LEO and FEO, are involved in circadian system(s) in mammals. Whereas the master LEO is located in the SCN of the hypothalamus [4], [5], localization of the FEO(s) still remains to be determined [6]–[8]. An output of a putative FEO is FAA, which can be assessed by restricted feeding (RF), daily limiting food availability to a restricted time window. SCN lesion studies demonstrated that FEO-driven FAA persists without the LEO, suggesting that FEO resides outside of the SCN [6], [9], [10]. It is also reported that FEO dominates LEO under RF in the entrainment of activity phase [4]. Moreover, disruption of the known circadian clock genes resulted in mostly partial or little alterations of FAA [11]. These data suggest that the FEO, which functions under limited nutrient availability, is a dominant circadian oscillator independent of LEO.

To elucidate molecular machinery driving circadian clock(s) in mammals, a list of candidate circadian clock/clock-controlled genes have been identified by microarray [8], [12], [13]. Among these was *Rgs16*, a member of RGS family. RGS proteins regulate G protein-coupled receptor (GPCR)-mediated signaling by negatively or positively interacting with downstream effectors [14]. In the brain, *Rgs16* is predominantly expressed in the SCN and thalamus [15], [16]. RGS16 exhibits robust circadian rhythms both in the SCN and in liver with its peak at 4–6 (zeitgeber time, ZT4-6) and 8–10 (ZT8-10) hours after light-on, respectively, suggesting that RGS16 is involved in the central and peripheral circadian clocks and/or their outputs [16]. Interestingly, a previous study demonstrated that *Rgs16* expression in liver was up-regulated during fasting and rapidly down-regulated by re-feeding [17]. In addition, expression of *Rgs16* in liver was restricted to periportal hepatocytes, the predominant locations of gluconeogenesis and lipolysis [17]. Considering that a number of GPCR ligands including vasoactive intestinal peptide (VIP; [18]), glutamate [19], [20], melatonin [21], [22], orexin [23], [24] and ghrelin [25], [26] have been implicated in the central or peripheral circadian clocks, these data raise the possibility that RGS proteins function in the circadian systems by modulating signaling through as yet unknown GPCR/GPCR ligand(s).

Here, to elucidate the possible role of RGS16 in the circadian clock *in vivo*, we generated two independent knockdown (KD) mouse lines using lentiviral vectors expressing two separate short hairpin RNAs (shRNAs) targeting the *Rge16* mRNA.

## Results

### Generation of the *Rgs16* knockdown mice

We designed different shRNAs against different regions of *Rgs16*. Each shRNA expression vectors was transfected into NIH3T3 cell line and levels of the *Rgs16* mRNA were quantified by quantitative RT-PCR (qPCR). The two shRNAs silencing endogenous *Rgs16* mRNA with the greatest efficiency (#41 and #53) were selected for producing two independent transgenic mouse lines (Fig. S2). We then produced separate high-titer lentiviral vector lots encoding the two shRNA expression cassettes as well as the GFP protein, and introduced them into fertilized one-cell stage mouse embryos by microinjection into the perivitelline space [27], [28]. The transgenic mice were selected by detecting GFP expression and the presence of the transgene confirmed by PCR-based genotyping.

### Expression of the *Rgs16* mRNA in the KD and wild-type brain and liver

**Figure 1:**
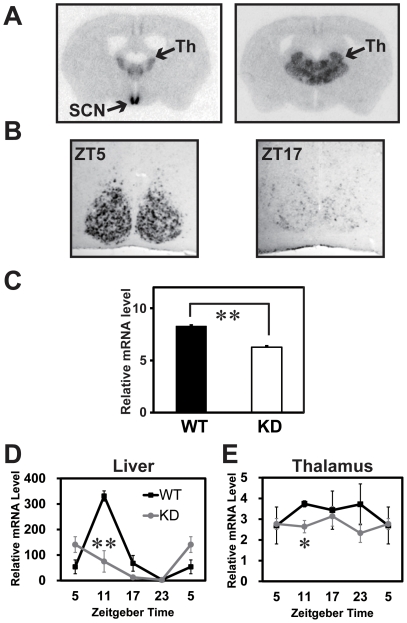
Spatial and temporal expression patterns of the *Rgs16* mRNA in brain and its reduction in the KD mice. **A, B,** *in situ* hybridization performed on brain sections. **A,** Specific and intense signals were observed in the SCN and thalamus (Th). **B,** Diurnal rhythms of the *Rgs16* transcript were observed in the SCN. **C,** ISH quantification of the *Rgs16* mRNA in the KD and WT SCN (n = 8 each) at ZT5. **P<0.01 vs. WT (Student's *t*-test). **D,** Expression of *Rgs16* in the KD and WT liver observed by qPCR. **P<0.01 vs. WT (Student's *t*-test). ##, *P*<0.01 circadian gene expression profile by 2-way ANOVA (WT vs. KD mice; n = 3–4). **E,** Expression of *Rgs16* in thalamus of the KD and WT mice (n = 3–4). *P<0.05 vs. WT.

We first examined the spacio-temporal expression patterns of the *Rgs16* mRNA in brain and liver of the KD and wild-type (WT) mice by *in situ* hybridization (ISH) and quantitative PCR (qPCR). In the WT brain, predominant expression of *Rgs16* was observed in the SCN, and at a lower level in thalamus (Fig. 1A). We also confirmed that *Rgs16* transcription exhibits robust circadian rhythms in the SCN (Fig. 1B). As shown in Fig. 1C, average *Rgs16* expression level was reduced the KD SCN (Fig. 1C). In the WT liver, robust circadian rhythms were observed peaking at ZT11 (Fig. 1D, F = 21.7, *P<*0.001). In the KD liver, average *Rgs16* expression level was reduced, and circadian changes were weakened (Fig. 1D, F = 6.06, *P<*0.05) relative to the controls (F = 21.7, *P<*0.001). In thalamus, no daily expression rhythms were observed in both WT (F = 0.65, *P>*0.05) and KD (F = 0.36, *P>*0.05) mice (Fig. 1E). The average expression level of *Rgs16* in the KD thalamus was lower than that of WT control, however, the knockdown efficiency was lower than that in liver (Fig. 1D, E).

### Free-running period of locomotor activity rhythm was shorter in the KD mice

**Figure 2:**
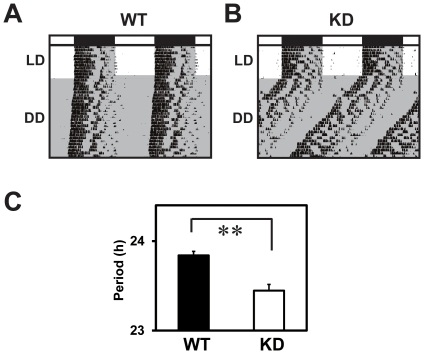
Short free-running period of locomotor activity rhythm in the KD mice. **A, B,** Locomotor activity rhythms of the WT (A) and KD (B) mice were entrained to 12∶12 LD. But in DD, the KD mice exhibited circadian period of locomotor activity shorter than that of WTs. The vertical axis in each graph indicates the day path. Horizontal open and closed bars indicate the light and dark periods, respectively. **C,** The difference in the circadian period between the KD and WT mice (n = 15 each) was statistically significant **P<0.01 vs. WT (Student's *t*-test).

To study the possible involvement of RGS16 in the central circadian clock, we examined locomotor activity rhythms of the KD and control mice. Wheel-running activities of individual mice were monitored under 12 hr light and 12 hr dark (LD) and constant dark (DD) conditions (Fig. 2A, B). In DD, The KD mice showed an average free-running period significantly shorter than that of the controls (23.84±0.05 hr in WT vs. 23.45±0.07 hr in KD, *P<*0.01, Fig. 2C). After locomotor activity rhythms were assessed, brains were sampled at ZT5 from individual KD and WT mice and quantitative ISH was performed on the brain sections. Average *Rgs16* mRNA level in the SCN was significantly decreased in the KD mice relative to controls (Fig. 1C).

### Total amount of locomotor activity was reduced in the *Rgs16* KD mice

**Figure 3:**
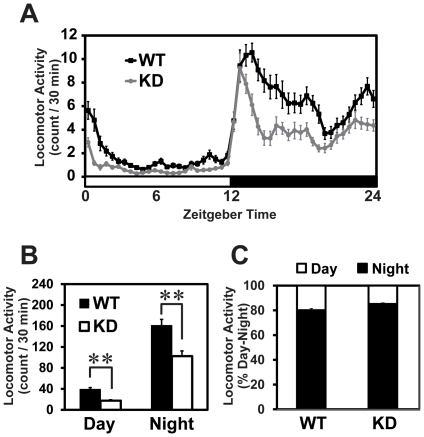
Reduced amount of locomotor activity in the *Rgs16* KD mice. **A,** Averaged daily activity plot of the KD and WT mice under LD condition. **B,** Average daytime or nighttime locomotor activity was significantly decreased in the KD mice as compared to that of WT controls (n = 21 each). **P<0.01 vs. WT (Student's *t*-test). **C** Averaged day/night ratio of locomotor activity was comparable between the KD and WT mice (n = 21 each).

We next compared total amount of locomotor activity of the *Rgs16* KD mice with that of controls. Total activity counts were assessed by an infrared sensor of KD and WT mice averaged every 30 minutes. The average activity of the KD mice was significantly lower than that of the controls regardless of day or night (Fig. 3A, B), whereas averaged day/night ratio of locomotor activity was comparable between the two genotypes (Fig. 3C).

### Attenuated FAA in the *Rgs16* KD mice

**Figure 4:**
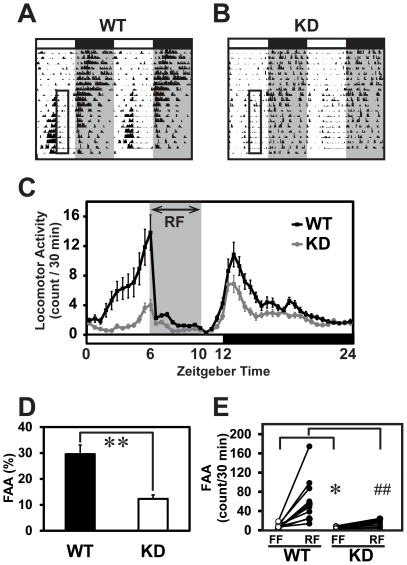
Attenuated FAA in the KD mice under daytime-RF. **A, B,** Examples of double-plotted actograms of WT (A) or KD (B) mice under RF. The RF time was set at ZT6–ZT10 (open box). Whereas WT mouse showed a strong FAA (A), those of KD were very weak (B). **C,** Averaged daily activity plot of the KD and WT mice under RF. **D,** Average percentages of FAA [100× activity counts (ZT2-6)/24-hour activity counts] during the last three days of RF between WT and KD mice were compared (n = 11 each). **P<0.01 vs. WT (Student's *t*-test). **E,** FAA counts of individual mice during 4 hours preceding RF (ZT2-6) averaged over the first 7 days of FF and the following 12 days of RF were compared in each genotype (n = 11 each). *P<0.05, vs. WT in FF; ##*P<*0.01 vs. WT in RF (Student's *t*-test).

The *Rgs16* mRNA in liver is not only circadianly regulated, but is also up-regulated by fasting and down-regulated by re-feeding [17]. These data raise the possibility that RGS16 is involved in metabolism in liver and in the food-driven behavior (FAA) regulated by a certain brain region(s) and/or liver. To test this, we next examined the effects of RF, daytime scheduled feeing for 4 hours (ZT6-ZT10), which is usually a resting period for nocturnal rodents. When mice are food-restricted during the day, FAA is observed about 3–4 hour prior to the feeding time [12]. In WT mice, a remarkable increase in the locomotor activities was observed for about 4 hours before the scheduled feeding time (Fig. 4A, C). On the other hand, the FAA of the KD mice was significantly attenuated in comparison with the controls (Fig. 4B, C). The reduction in the average percent FAA in the KD animals compared to that in the controls was statistically significant (Fig. 4D, *P* *<*0.01). Fig. 4E demonstrates FAA counts of individual KD and WT mice under RF as compared with baseline activity counts under free feeding (FF, as measured activity during ZT2-6). Bouts of locomotor activity during daytime was strongly reduced in KD group by RF, however, those in nighttime were comparable between WT and KD mice under FF or RF (Compare Fig. 3A and 4C). It is of note that, under the RF schedule, both WT and KD mice obtained almost the same amount of food during ZT6-ZT10 (0.88±0.08/10 g body weight/day for the WT; 0.80±0.05/10 g body weight/day for KD). Although body weight was slightly reduced by RF in both WT (33.1±2.8 g prior to and 30.0±1.6 g after RF) and KD mice (30.7±1.6 g prior to and 27.6±0.8 g after RF), there was no significant difference in the amount of food intake or the body weight between the two genotypes (*P*>0.05, Student's *t*-test).

### Daytime-RF phase-advanced the *Per2* mRNA rhythm in WT, but not in KD

**Figure 5:**
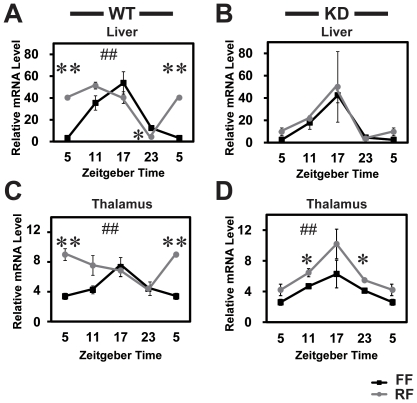
Daytime-RF phase-advanced circadian rhythms of *Per2* in liver and thalamus in WT, but not in the KD mice. Under RF (ZT6-10), the WT mice liver (**A**) and thalamus (**C**) phase-advanced *Per2* rhythms by 6 hours and 12 hours, respectively, as compared to the control mice under FF. In contrast, the circadian phase of *Per2* in the KD mice was not shifted in either liver (**B**) or thalamus (**D**) by RF. ##*P<*0.01 circadian gene expression profile by 2-way ANOVA (FF vs. RF mice, A, C, D). **P<0.01, *P<0.05 vs. WT (Student's *t*-test). n = 3–4 for each ZT point. FF, free feeding; RF, restricted feeding.

Reduced FAA has been reported in several knockout mice, but the mechanism underlying such behavioral change remains to be elucidated. To examine whether RF elicits FAA by affecting expression profiles of circadian clock gene and/or *Rgs16* in brain and liver, we sampled KD and WT mouse tissues at 4 time points (ZT5, 11, 17, and 23) and performed qPCR. Fig. 5 demonstrates the circadian rhythms in the *Per2* clock gene expression in liver and thalamus in both the WT and the KD mice, where *Rgs16* transcripts are abundantly expressed. Under FF, the *Per2* mRNA exhibited circadian rhythms both in liver (F = 21.7 *P<*0.01 for WT, F = 16.1 *P<*0.05 for KD, one-way ANOVA) and thalamus (F = 5.1 *P<*0.05 for WT) with a peak at ZT17 in both genotypes. Under RF in WTs, the *Per2* rhythms were significantly phase-advanced, peaking at ZT11 and ZT5 in liver and thalamus, respectively (Fig. 5A, C; F = 25.2 *P<*0.01 for liver, F = 4.1 *P<*0.05 for thalamus). In contrast, the phase of the *Per2* rhythms in the KD mice was unaffected by RF either in liver or thalamus (Fig. 5B, D).

## Discussion

In the present study, we generated transgenic *Rgs16* KD mice using lentiviral vectors carrying a shRNA expression cassette and a modified microinjection method, i.e., perivitelline injection [27], [28]. This gene silencing technique enabled us to efficiently generate sufficient numbers of transgenic mice within a few months, because of the higher rate of transgenesis by lentiviral vector injection compared with the conventional pronuclear DNA microinjection [29]–[31]. Moreover, this *in vivo* KD strategy could be a method of choice when disruption of the gene of interest is likely to cause lethality *in vivo*, or leads to a lack of phenotype because redundant functionalities from within the gene family or from related gene(s) result in compensation for the gene loss. We anticipated that *Rgs16* knockout could potentially be compensated by other *Rgs* genes (*Rgs2* and/or *Rgs4*), because these genes, which belong to the same R4 subfamily along with *Rgs16*, are also circadianly expressed in the SCN [16]. Consistent with this expectation, another group reported no significant phenotype in the *Rgs16* knockout mice [32].

Our study provided evidence that G protein signaling modulated by RGS16 plays a role not only in the master LEO, but also in the FEO(s). Previous studies have indicated potential circadian roles of several GPCRs in the SCN. For instance, knockout and other studies strongly suggest that VIP and its receptor VPAC2, a member of GPCR family, are important for synchronization of the circadian clock and for sustaining circadian rhythmicity [18]. Likewise, GPCR ligands/GPCRs such as orexin/receptor and ghrelin/receptor are also involved in the FEO [23]–[26]. Further studies to identify upstream GPCR(s) will shed light on a significant role(s) of RGS16 in the SCN, thalamus and liver.

In the present study, expression level of *Per2* was up-regulated by RF, consistent with the previous report demonstrating an RF-induced change in basal level of gene expression [33]. Food seeking/satiety and energy metabolism may be one of the factors for FAA formation [23], [24], [34]. In addition, previous studies have shown that the volume of food can produce large phase-shifts in the FAA rhythm in rats, and also large phase-advance in the *Per2* gene expression in mouse liver in a food volume-dependent manner [35]. In our data, food availability and body weight change during RF conditions were not significantly different between the WT and KD mice, suggesting that deficit of FAA formation and phase-shift of the *Per2* expression in liver did not lead to impairment of food seeking ability and/or the digestive system. RGS16 is the first molecule that has been reported to control not only FAA formation but also phase-shift of liver clock by RF. Thus, our data could provide a new insight into where and how these signaling molecules including RGS proteins affect the circadian timing systems.

Our present study also demonstrated that, whereas the circadian rhythms of the *Per2* mRNA were significantly phase-advanced by daytime-RF, both in liver and thalamus compared to free-feeding WT controls, the *Per2* rhythms in the KD mice did not phase-shift in either organ. These results suggest that RGS16 is potentially an upstream regulator of the *Per2* rhythms in liver and thalamus, whereas expression of *Rgs16* itself is regulated by the circadian clock. The data also suggests that RGS16 is involved in the phase-shift of both behavioral and molecular rhythms evoked by RF. It is noteworthy that a phase-advance in the *Per2* rhythms by daytime-RF was observed in thalamus. These data, together with the specific expression of *Rgs16* in this brain region, imply that thalamus plays a role in the FEO. It has been reported that complete paraventricular thalamic nucleus (PVT) lesion affects LEO, but not FEO [36], although involvement of thalamic regions other than PVT in the circadian systems needs to be determined. Interestingly, the report also indicated that PVT ablation increased total daily activity, suggesting that this thalamic region is involved in regulating behavioral output. Altogether, our data along with previous studies raise a possibility that RGS16 in thalamus modulates light-entrained and/or food-entrained behavior by regulating activity levels and/or circadian behavioral rhythms under different environmental conditions.

Our study provided for the first time evidence suggesting that two distinct regions of the body, thalamus and liver, where *Rgs16* mRNA is abundantly expressed, are involved in the regulation of the FAA under restricted food availability. The data raise the question of whether liver or thalamus plays a lead role in the FEO-operated FAA, or both of these regions regulate the behavior, either independently or cooperatively. Further studies in search of upstream GPCRs/ligands and downstream signaling molecules will elucidate the mechanism of how RGS16 specifically regulates food-entrained behavior in different loci.

## Materials and Methods

### Animals

C57BL/6J inbred mice were purchased from CREA Japan and used for generating transgenic mice and all other experiments. Animals were provided with food and water *ad libitum*, and entrained to 12 hr light: 12 hr dark condition (12∶12 LD) for at least 2 weeks at 23±2°C, All animals were cared for in accordance with the Law (No. 105) and Notification (No. 6) of the Japanese Government, and all experiments were conducted under permission of the Experimental Animal Welfare Committees of Kinki University (Permission #KDMS-16-002) and Waseda University (Permission #08A36).

### shRNA

Six shRNAs were designed from the *Rgs16* cDNA sequence, and annealed double-stranded oligomers were subcloned into the pSilencer 1.0-U6 siRNA Expression Vector (Ambion). Knockdown efficiencies of the expression vectors carrying individual shRNAs were evaluated by transient transfection of the vectors into mouse NIH3T3 (RIKEN Cell Bank) fibroblast cell line by Lipofectamine 2000 (Life Technologies) according to manufacturer's instruction. After 48 hours of incubation, cells were lysed using Sepazol-RNA I (Nacalai Tesque), RNA was extracted, and quantitative PCR (qPCR) was performed using *Rgs16* primers. Two shRNAs (#41, 53), which significantly suppressed the endogenous *Rgs16* expression levels, were used for transgenesis. The sequences of annealed oligonucleotides for expressing #41 or #53 shRNA were as follows:

#41: 5′-GCGAGGAGTTCAAGAAGATTTCAAGAGAATCTTCTTGAACTCCTCGCTTTTTT-3′ and 5′-AATTAAAAAAGCGAGGAGTTCAAGAAGATTCTCTTGAAATCTTCTTGAACTCCTCGCGGCC-3′

#53: 5′-GAGAACTGACCAAGACAAATTCAAGAGATTTGTCTTGGTCAGTTCTCTTTTTT-3′ and 5′-AATTAAAAAAGAGAACTGACCAAGACAAATCTCTTGAATTTGTCTTGGTCAGTTCTCGGCC-3′

### Lentiviral vector construction and production of high-titer lentiviral vector particles

The two shRNA sequences (#41 and #53) targeting *Rgs16* along with the mouse U6 promoter were PCR-amplified from the pSilencer vectors and cloned into the pTZV TranzVector™ (Tranzyme) as depicted in Fig. S1. An eGFP fluorescent marker driven from the human immediate early cytomegalovirus virus (CMV) promoter is co-expressed with the shRNA hairpin sequence. High-titer lentiviral vector particles were generated using the Trans-Lentiviral™ Vector Packaging system [37]. Viral particles were concentrated by ultracentrifugation. Functional titers were determined by transducing HEK293T (ATCC) cells with limiting dilutions of virus and counting GFP-positive colonies.

### Generation of *Rgs16* KD mice

Fertilized oocytes were harvested from super-ovulated C57BL/6J female mice. Viral vectors at a concentration of 1×10^9^/ml were microinjected into the perivitelline space of the oocytes using a FemtoJet injector (Eppendorf), and then reimplanted into the oviduct of pseudo-pregnant recipient females after 2–4 hours of microinjection. Injections were performed under 400× magnification (DM IRE2, Leica), and injection volume was approximately 100 nl.

### Genotyping and evaluation of the KD mice

For genotyping, genomic DNA was extracted from a tail tip of each mouse by a standard protocol using proteinase K and following phenol/chloroform extraction. Using the genomic DNA as a template, PCR was performed using GFP primers. To confirm expression of the GFP protein, we observed tail tips under fluorescent microscopy.

### qPCR

Total RNA was extracted from tail tip, liver, and thalamus of each KD or WT control mouse using RNeasy Mini Kit (Qiagen). The extracted RNA was reverse-transcribed into cDNA using High Capacity cDNA Reverse Transcription Kit (Applied Biosystems). For qPCR reaction, SYBR GREEN Premix ExTaq (Takara) was used following manufacturer's instruction. The sequences of primers used in this study are as follows. GFP primers: 5′-AGCAAAGACCCCAACGAGA-3′ and 5′-GGCGGCGGTCACGAA-3′; *Rgs16* primers: 5′-TGCTTGTGAACAGGGCTAACTG-3′ and 5′-CTCCCTCCTTAGACCCCATCTT-3′; *Per2* primers: 5′-TGTGTCTTACACGGGTGTCCTA-3′ and 5′-ACGTTTGGTTTGCGCATGAA-3′, 18S rRNA primers: 5′-CGGCTACCACATCCAAGGAA-3′, 5′-GCTGGAATTACCGCGGCT-3′.

The value of the PCR product of the target gene was normalized to that of 18S rRNA.

### Measurement of locomotor activity rhythm

Mice were individually housed in translucent polypropylene cages under 12∶12 LD (200 lux) and DD, and locomotor activity was assessed either by a running wheel (Fig. 2) or an area sensor (F5B, Omron; Fig. 3, 4). Activity was continuously monitored and analyzed using ClockLab software (Actimetrics).

### Schedules for RF

Individually housed KD mice and WT controls were maintained in a 12∶12 LD cycle under FF for at least 7 days. The RF experiment for the measurement of anticipatory activity was performed as previously described [38]. Briefly, after 1 day of food deprivation starting at ZT12 (day 0), food was restricted to ZT6-ZT10 for 12 days (day 1–12). From day 13 to 14, food was again withdrawn for the entire day to record motor activity under food deprivation. Another RF experiment for the measurement of clock gene expression was performed. After application of the same RF conditions, animals were sacrificed at ZT5, 11, 17, and 23 on day 13 under food deprivation.

### *In situ* hybridization (ISH)

Riboprobe was labeled with ^35^S-UTP (Amersham/GE Healthcare) by *in vitro* transcription using either T7 or SP6 polymerase (Promega). Frozen mouse brain sections (40 µm thickness) were hybridized with riboprobe for overnight and apposed to Kodak film (BioMax MR). The *Rgs16* cDNA (entire ORF length 606 bp) was amplified by PCR and subcloned into pGEM-T Easy Vector (Promega). The plasmids were linealized with *Nco*I to synthesize riboprobe.

### Statistics

Results were expressed as the mean ± SEM. One-way ANOVA was applied to evaluate significant difference of circadian rhythmicity of gene expression, and 2-way ANOVA was applied to evaluate significance between KD and control mice. The significance of the differences between groups was determined by the Student's *t*-test. Statistical analysis software (StatView version 5.0, SAS Institute) was used.

## Supporting Information

Figure S1 **Schematic representation of the shRNA lentiviral vectors.** The shRNA hairpin sequences (#41 and #53) targeting mouse *Rgs16* is cloned into pTZV, a HIV1-based self-inactivating (SIN) lentiviral transfer vector containing the central polypurine tract/termination sequence (FLAP) and the Woodchuck hepatitis virus post-transcriptional regulatory element (WPRE) for enhanced gene expression. A PCR-amplified fragment containing the mouse U6 promoter, shRNA hairpin, and pol III termination sequence (TTTTT) is cloned into pTZV between 5′ – *Cla*I and 3′ – *Bam*HI sites. A GFP marker is co-expressed with the shRNA from a CMV promoter to enable visualization of transduced cells and transgenic embryos.
(EPS)

Figure S2 ***In vitro*** **knockdown of the** ***Rgs16*** **mRNA by shRNAs used in this study.** 2 µg of the pSilencer 1.0 vector expressing either of the two shRNAs (#41 or #53) was transfected into the NIH3T3 mouse fibroblast cell line with Lipofectamine 2000. RNA was extracted from each of the transfected cells two days after the transfection, and expression levels of *Rgs16* were compared by qPCR (see Materials and Methods). Both #41 and #53 shRNAs significantly reduced the average *Rgs16* mRNA level (**P<0.01, n = 4).
(EPS)
